# Gene essentiality in cancer is better predicted by mRNA abundance than by gene regulatory network-inferred activity

**DOI:** 10.1093/narcan/zcad056

**Published:** 2023-11-28

**Authors:** Cosmin Tudose, Jonathan Bond, Colm J Ryan

**Affiliations:** Systems Biology Ireland, University College Dublin, Dublin, Ireland; School of Medicine, University College Dublin, Dublin, Ireland; The SFI Centre for Research Training in Genomics Data Science, Ireland; Systems Biology Ireland, University College Dublin, Dublin, Ireland; School of Medicine, University College Dublin, Dublin, Ireland; Children's Health Ireland at Crumlin, Dublin, Ireland; Systems Biology Ireland, University College Dublin, Dublin, Ireland; School of Computer Science, University College Dublin, Dublin, Ireland; Conway Institute, University College Dublin, Dublin, Ireland

## Abstract

Gene regulatory networks (GRNs) are often deregulated in tumor cells, resulting in altered transcriptional programs that facilitate tumor growth. These altered networks may make tumor cells vulnerable to the inhibition of specific regulatory proteins. Consequently, the reconstruction of GRNs in tumors is often proposed as a means to identify therapeutic targets. While there are examples of individual targets identified using GRNs, the extent to which GRNs can be used to predict sensitivity to targeted intervention in general remains unknown. Here we use the results of genome-wide CRISPR screens to systematically assess the ability of GRNs to predict sensitivity to gene inhibition in cancer cell lines. Using GRNs derived from multiple sources, including GRNs reconstructed from tumor transcriptomes and from curated databases, we infer regulatory gene activity in cancer cell lines from ten cancer types. We then ask, in each cancer type, if the inferred regulatory activity of each gene is predictive of sensitivity to CRISPR perturbation of that gene. We observe slight variation in the correlation between gene regulatory activity and gene sensitivity depending on the source of the GRN and the activity estimation method used. However, we find that there is consistently a stronger relationship between mRNA abundance and gene sensitivity than there is between regulatory gene activity and gene sensitivity. This is true both when gene sensitivity is treated as a binary and a quantitative property. Overall, our results suggest that gene sensitivity is better predicted by measured expression than by GRN-inferred activity.

## Graphical Abstract

**Graphical Abstract:**
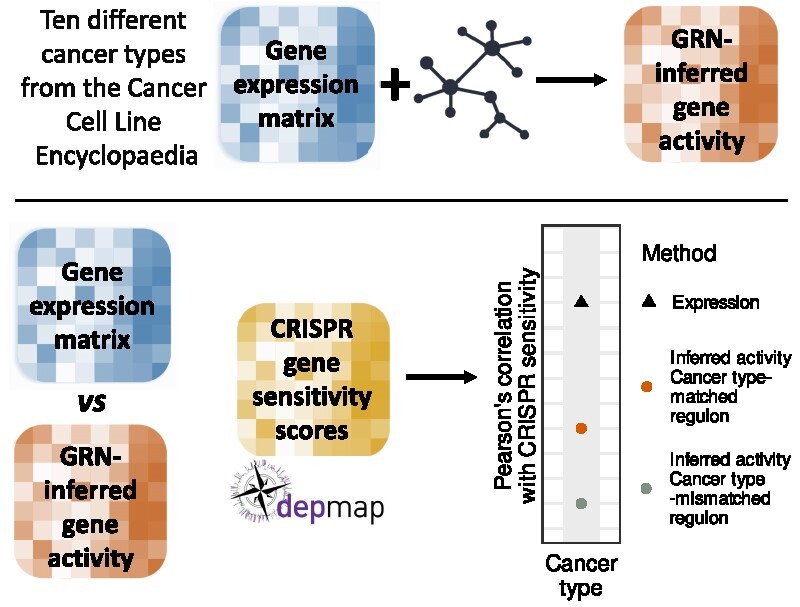

## Introduction

A large volume of cancer molecular profiles have become available through compendia such as The Cancer Genome Atlas (TCGA) and the Cancer Cell Line Encyclopedia (CCLE) (1). Additionally, maps of cancer vulnerabilities have been generated using CRISPR and drug screens through efforts such as The Cancer Dependency Map (DepMap) and the Genomics of Drug Sensitivity in Cancer (GDSC) (2–7). A major outstanding challenge is to identify therapeutic targets for molecularly defined cohorts.

Many genetic alterations drive oncogenesis by altering transcriptional programs that govern critical cellular processes such as proliferation, cell cycle and apoptosis via gene regulatory networks (GRNs) (8). In cancer, GRN perturbation disrupts key transcriptional programs, and can lead to changes in response or resistance to therapies (9,10). Targeting GRNs to restore normal cell function is a clinically attractive idea. Currently, there are ongoing trials targeting molecular networks, such as STAT3/5 or menin in acute myeloid leukemia (AML), estrogen receptor in ER+/HER2- breast cancer and MDM2 as part of the p53-MDM2 interaction in various cancer types (11).

Computational tools are often employed to reconstruct GRNs with a view to identifying therapeutic targets (12,13), e.g. ARACNe (14), GENIE3 (15) and KBoost (16) interpret correlations from transcriptomes to construct GRNs. Each GRN is composed of regulons and each regulon contains a regulatory gene, its targets, and the weights between. These GRNs, however, are *in-silico* inferred, and biological validation is not straightforward. This is particularly challenging in human cells, as a gold standard map of human GRNs does not yet exist.

Transcription factor (TF) activity, also referred to as protein activity (17) or regulon activity (18), represents the inferred activity of a regulatory gene derived from the variance in transcript abundance of its targets, according to a pre-determined regulon (19). Inferred activity has been used to investigate drug response (20,21), uncover ‘hidden’ drivers (22) and showcase the role of ‘master regulators’ in cancer (17,23,24). However, validation often involves assessing the impact of perturbing a small number of example genes and measuring the resulting transcriptional changes (17). Previous work has assessed the extent to which inferred activity associates with mutational status and copy number variation (21,25). Somewhat surprisingly, in a large multi-omic dataset of tumor and cell line samples, Sousa *et al.* found limited correlation between copy number variation and the inferred activity of transcription factors (25). While the mutation of individual genes (e.g. *TP53*, *GATA6*) could in some cases be associated with altered activity of the encoded transcription factor, this was not the default. In general, the authors observed a lower correlation between inferred activity and protein abundance than they observed between inferred activity and mRNA abundance (25). This is somewhat surprising as one would assume that the protein abundance of a regulatory gene is a better proxy for its activity than its mRNA abundance. Akin to GRN inference methods, there are many activity inference methods, with little consensus across them (26).

Given that GRNs have been suggested to drive oncogenic processes, dysregulation of regulatory gene activity may lead to vulnerability to perturbation and dependency to regulatory genes (27,28). However, this has not been assessed at a systematic level. Here, we used CRISPR screens as a precise method to validate whether GRN-inferred activity can predict sensitivity to inhibition. In CRISPR screens, each gene is perturbed with sgRNAs, and a gene's sensitivity to inhibition is assigned a score calculated from cell growth and survival (29). The DepMap project uses this approach to characterize the gene sensitivity profiles of more than 1,000 cell lines via genome-wide CRISPR screens (2–5). We inferred regulatory gene activity in these cell lines using both computationally derived and curated regulons (30). We then evaluated correlations between gene sensitivity and inferred activity across cell lines. Additionally, in regulatory genes, we compared expression and activity in their ability to predict sensitivity to inhibition. Although gene essentiality is often discussed as a binary property, processed CRISPR screens typically report a quantitative score representing the sensitivity of each cell line to the inhibition of each gene. In this work, we analyze the ability of GRN-inferred activity, and mRNA expression, to predict sensitivity to gene inhibition as a quantitative property and also as a binary score (essential / non-essential). Overall, we found little evidence of activity estimation methods providing an advantage over measured mRNA abundance.

## Materials and methods

### ARACNe regulons processing

We loaded cancer type-specific ARACNe regulons from the aracne.networks 1.20.0 R package and transformed them into data frames using the ‘reg2tibble’ function from the binilib 0.2.0 R package. We converted Entrez IDs into gene symbols via the org.Hs.eg.db 3.14.0 R package. We calculated an updated mode of regulation (MOR) by multiplying the likelihood with the sign of the MOR for each interaction. MOR indicates the directionality of the interaction (i.e. −1 = inhibition; 1 = activation). ‘Source’ genes missing in the expression data were filtered out.

To infer ARACNe regulons from the BRCA and LUAD CCLE gene expression data, we ran ARACNe-AP with the default settings (14).

### GRNdb regulons processing

We downloaded TCGA-inferred cancer type-specific regulons from http://www.grndb.com (31). We used the gene symbols provided. These regulons contain a weight calculated with GENIE3 (15), but do not contain directionality (inhibition or activation), which the ARACNe regulons do, by providing MOR. Therefore, we inferred MOR using the TCGA dataset, as it was used to build the regulons, as follows:

We downloaded log_2_(tpm + 1) normalized RNA-Seq from the UCSC Treehouse Public Data, v11 Public PolyA. We separated the transcriptomics into ten matrices, for each cancer type, using the ‘disease’ column from the clinical data file. For each cancer type, we removed genes with 0s in more than half of the samples, whilst we imputed the others using ‘impute.knn’ from the impute 1.68.0 R package. We inferred the MOR for each interaction in the GRNdb regulons by calculating the Spearman correlation between the expression of each regulatory gene and its target. We computed an updated MOR by multiplying the GENIE3 weight with the sign of the MOR from the previous step.

To build GRNdb-like regulons from the CCLE, we followed the instructions from Fang *et al.* (2021) (31).

### DoRothEA regulons processing

We loaded the human DoRothEA regulons from the dorothea 1.6.0 R package (30). For the downstream analysis we used the high confidence regulons: A, B and C.

### Data wrangling

**Figure 1.**
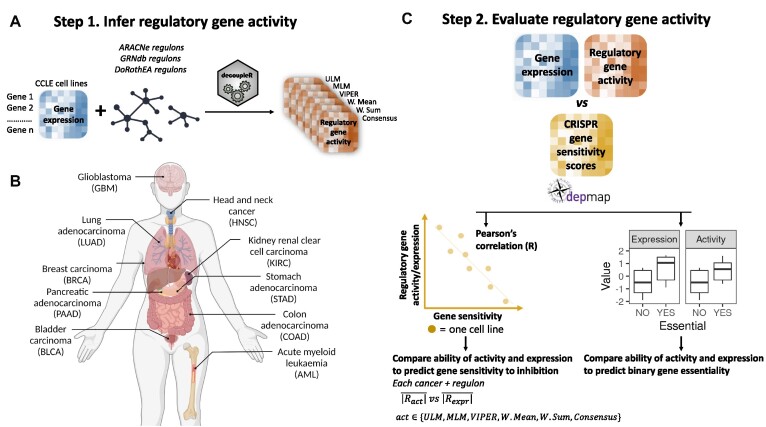
Workflow for evaluating TF activity estimation using CRISPR gene sensitivity profiles from DepMap. (**A**) The activity of regulatory genes was inferred in cancer cell lines using six methods from the DecoupleR package. Gene expression profiles from the CCLE were paired with cancer type-specific regulons from ARACNe, GRNdb and curated pan-cancer regulons from DoRothEA to infer regulatory gene activity. (**B**) Ten different cancer types (TCGA abbreviations in brackets) with CCLE gene expression profiles and regulons were used for the analysis (generated using BioRender.com). (**C**) Activity inferred using different GRNs and different activity estimation methods was compared with gene expression using two approaches 1. The Pearson's correlation between inferred activity and gene sensitivity was compared with the Pearson's correlation between expression and gene sensitivity. 2. Activity and expression were used to look at the degree of separation between essential and non-essential genes in a binary fashion using the common-language effect size (CLES) and a Wilcoxon test.

We downloaded CCLE gene expression, gene sensitivity data and cell line information from DepMap release 21Q4 (2–5). We filtered these for cell lines from cancer types present in Figure 1B and for the downstream analysis we kept only cell lines present in both gene expression and gene sensitivity datasets. Likewise, only genes present in both datasets were retained for each cancer type. For gene nomenclature we used HGNC symbols, discarding Entrez IDs. For each cancer individually, we dropped genes with >20% 0s across samples in the gene expression profile.

### Filtering 'sometimes’ essential genes

We defined genes as essential in a given cell line if they had a CHRONOS score <−0.6 in that cell line. Within each cancer type, we restricted our analysis to genes that were variably essential across cell lines from that cancer type (i.e. genes that were either essential in all cell lines or non-essential in all cell lines were filtered out). Therefore, we were left with sometimes-essential genes only, in order to study variation in sensitivity to inhibition across tumor cells. The CHRONOS score is a scoring system for quantifying and normalizing outputs from CRISPR screens and aggregating the results of multiple gRNAs targeting the same gene into a single gene-level score. Full details about CHRONOS are available in Dempster *et al.* (2).

For the analysis across all cancers (pan-cancer) we implemented three different thresholds: 1% (i.e. nine cell lines), 5% (i.e. 47 cell lines) and 10% (i.e. 97 cell lines). This means for a gene to be considered sometimes essential it had to be essential in at least 1% (or 5% or 10%) of cell lines and non-essential in at least 1% (or 5% or 10%, respectively) of cell lines.

### Computing regulatory gene activity

We ran the ‘decouple’ function from the decoupleR (32) 2.1.8 package individually on each cancer expression matrix paired with each regulon (see Figure 1). DecoupleR infers activity via five different methods: Univariate Linear Model (ULM), Multivariate Linear Model (MLM), Virtual Inference of Protein-activity by Enriched Regulon (VIPER), Weighted Mean (W. Mean), Weighted Sum (W. Sum) activity. It then calculates a Consensus across all methods. We used ARACNe, GRNdb and DoRothEA regulons.

### Correlation analysis

For each gene we calculated the Pearson's correlation between inferred regulatory gene activity/expression and gene sensitivity scores for sometimes essential genes.

We filtered for genes with a significant Pearson's correlation (*P* < 0.05) and grouped in categories based on absolute Pearson's R: 0.2, 0.4, 0.6, 0.8, 1 and based on the sign of R: positive or negative. We used the cor.test function from the R stats 4.1.2 package.

### Regulon size stratification

To stratify our analysis based on regulon size we separated the regulons into three categories based on the number of targets the regulon regulates: small (≤20 targets), medium (>20 targets & ≤100 targets) and large regulons (>100 targets). This could only be performed on GRNdb and DoRothEA regulons, as ARACNe regulons are provided with edge values between every regulatory gene-target pair combination.

### Regulon stratification based on the number of unique targets

To stratify our analysis based on the number of unique targets each regulon regulates we separated the regulons into three categories: No unique targets, ≤10% of targets are unique and > 10% of targets are unique. A target is unique in a regulon if it is not present as a target gene in any other tested regulon. For the same reasons stated above, this could only be performed on GRNdb and DoRothEA regulons.

### Enrichment analysis

We ran Gene Ontology (GO) term enrichment analysis using the WebGestalt R package, with FDR = 10% (33).

We used the genes marked as ‘oncogene’ in the cancer gene census (CGC, https://cancer.sanger.ac.uk/census) (34) to test for oncogene enrichment in the genes where activity is better correlated with sensitivity, using a Fisher's exact test. Similarly, we tested for master regulator enrichment using the master regulator list from Paull *et al.* (2021) in Supplementary Table S2 (35).

We have performed GO enrichment analysis on genes with an absolute correlation between Consensus activity/expression and sensitivity to inhibition larger than 0.6. We have looked at the overlap of enriched GO terms between all GRN methods and expression.

### Calculate per gene variance for each method

For each gene we calculated the variance across all samples, all regulon sources and cancer type + cancer type-matched regulons.

### Comparing activity methods

For each possible cancer type + cancer type-matched regulon combination we calculated the mean Pearson's correlation across all genes (no p-value filtering) for each activity method and compared.

We fit linear models using the lm function from the R stats package (v4.1.0):

- |R| ∼ Cancer type + Regulon source + Activity method + No. cell lines + RNA-Seq variance
- |R| ∼ Cancer type + Regulon source + Activity method + No. cell lines + No. Unique targets
- |R| ∼ Cancer type + Regulon source + Activity method + No. cell lines + Regulon Size

The former two linear models were fit only using GRNdb and DoRothEA regulons. This was because in ARACNe regulons all regulatory genes have an edge with every possible target in the genome, leading to all regulons being the same size and having no unique targets.

We estimated the percentage of the variance each term in the linear model explains using adjusted R-squared.

### Comparing regulons

For each possible cancer type + regulon combination, we calculated the mean Pearson's correlation across all genes (no p-value filtering) for each activity method to investigate whether cancer type-matched regulons are more predictive of sensitivity than mismatched regulons. We plotted the absolute mean Pearson's R for each combination and assigned ranks 1–10 for each cancer. We conducted Unpaired Two-Samples Wilcoxon tests (wilcox.test function from the R 4.1.2 stats package) to compare the ranks of cancer type-matched regulons to the ranks of cancer type-mismatched regulons.

### Common language effect size calculation

For each cancer type + cancer type-matched regulon combination, we calculated the common language effect size (CLES) across sometimes essential genes for each activity method. Here we considered genes essential in at least three cell lines, and non-essential in at least three cell lines as sometimes essential. We used the CLES function from the bmbstats v0.0.0.9001 R package to predict binary essentiality.

We filtered for significance based on the expression/activity difference in non-essential vs. essential genes (Wilcoxon unpaired test *P* < 0.05) and counted the number of genes for each method with a CLES >0.7, >0.8 and >0.9, respectively.

R code to run analyses is available at: https://github.com/cancergenetics/GRN_activity_corr_essentiality.

## Results

### Variation in correlation between activity and gene inhibition sensitivity is driven more by cancer type than activity estimation method

Estimating regulatory gene activity requires a GRN (containing edges between regulatory genes and targets) and a gene expression matrix (quantifying the expression levels of all genes in a set of samples) (Figure 1A) (32). Typically, activity estimation methods assign an activity score to a given regulatory gene such that higher expression of the gene's targets in a given sample is associated with a higher activity score for the regulatory gene in that sample. Here, rather than focusing on a single GRN or single activity estimation method, we assessed three GRN sources and six activity estimation methods (Figure 1A).

We selected ARACNe (23,36), GRNdb (15,31) and DoRothEA (30) as representatives of different GRN reconstruction approaches—ARACNe is one of the longest-established methods and infers GRNs solely from transcriptomes; GRNdb is more recently developed and uses GENIE3 GRNs inferred from transcriptomes that are further refined with ChIP-Seq data; while DoRothEA contains curated GRNs that incorporate cis-regulatory information from ChIP-Seq peaks, literature curated resources, and TF binding motifs within promoters. For ARACNe and GRNdb, we obtained cancer type-specific GRNs, i.e. a breast cancer GRN was assembled from gene expression profiles of breast cancer samples, while for DoRothEA, only cancer type-agnostic GRNs were available.

**Figure 2.**
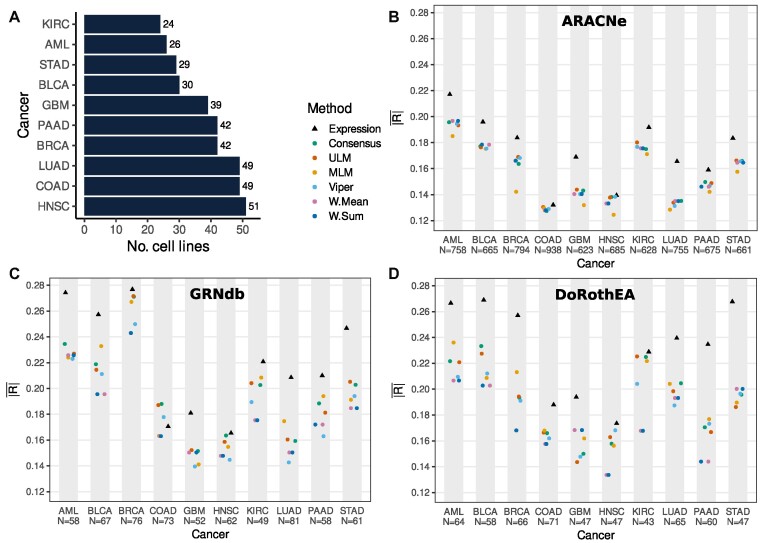
Activity estimation methods have similar performance in predicting gene sensitivity. (**A**) Number of cancer cell lines present in DepMap and CCLE for each cancer type. (**B–D**) Comparison between the different inferred activity methods (paired with cancer type-matched regulons) correlating with gene sensitivity and gene expression correlating with gene sensitivity. (**B**) ARACNe. (**C**) GRNdb. (**D**) DoRothEA (*N* = number of regulatory genes used to generate $\overline {| {\boldsymbol{R}} |}$ for each cancer type).

Using decoupleR, we estimated activity in the DepMap cancer cell lines using five different methods: ULM, MLM, VIPER, W. Mean, W. Sum as well as a consensus score calculated by decoupleR using all five scores. These methods work in similar ways: they estimate enrichment scores for each regulatory gene based on its number of targets, targets’ expression, and MOR (i.e. inhibition or activation). We then determined the mean absolute Pearson's correlation ($\overline {| R |}$) between the regulatory gene activities and CRISPR gene sensitivity scores across cell lines from specific cancer types. Cancer types for which we had matched GRNs derived from relevant TCGA tumor samples were included in this analysis, resulting in ten cancer types being assessed (Figure 2A). The number of cell lines for each cancer type ranged from 24 (kidney renal clear cell carcinoma - KIRC) to 51 (head and neck squamous cell carcinoma - HNSC) (Figure 2A). For comparison, we also included the $\overline {| R |}$ between mRNA abundance and gene sensitivity scores.

We used absolute correlation to assess the association between regulatory gene activity/mRNA abundance and sensitivity because we anticipated that both increased activity (e.g. resulting from amplification) and decreased activity (e.g. resulting from copy number loss) might result in increased sensitivity to inhibition. The former might occur with oncogene addiction-like effects, e.g. *MYC* amplification driving *MYC* sensitivity, while the latter might occur with haploinsufficiency-like effects, e.g. reduced copy number or expression/activity of a gene making cells more sensitive to further perturbation of that gene (37,38).

Correlations were only calculated for genes that were (i) identified as regulatory genes in the GRN and (ii) identified as essential in a subset of cell lines from the cancer type assessed, i.e. after excluding genes that are always or never essential (see Materials and methods). A consequence of these criteria is that different GRN methods were evaluated over different gene sets, because they include different regulatory genes (e.g. the ARACNe breast cancer network contains 6,054 regulatory genes, while DoRothEA only contains a total of 271 regulatory genes).

Across all cancer types, the six activity scores yielded an $\overline {| R |}$ between 0.12 and 0.28 for GRNdb, ARACNe and DoRothEA (Figure 2B–D). Across all cancer types and activity estimation methods, GRNdb shows the highest average correlation ($\overline {| R |}$ = 0.189), followed by DoRothEA (0.185) and ARACNe (0.156). However, the genes included in each GRN varied significantly – between 628 and 938 genes assessed for ARACNe, between 49 and 81 for GRNdb, and between 43 to 71 for DoRothEA (Figure 2B–D). Summarizing over all regulon sources and cancer types, Consensus had the highest correlation with gene sensitivity, with an $\overline {| R |}$ of 0.181. W. Sum and W. Mean performed identically and were jointly the lowest performing of the methods ($\overline {| R |}$ = 0.171).

Although there are differences in $\overline {| R |}$ between the different activity estimation methods and the different GRN sources, visual inspection of the results in Figure 2 suggests that cancer type may have a much bigger influence than either activity method or GRN source. For instance, although there is variation between the $\overline {| R |}$ calculated with different activity estimation methods using ARACNe regulons in AML (range 0.18–0.2) there is a much bigger difference between the $\overline {| R |}$ calculated in AML and GBM (median 0.195 for AML; 0.14 for GBM). To understand the relative contributions of different factors to the variability in $\overline {| R |}$, we fit a linear model with three terms: cancer type, regulon source (ARACNe, GRNdb, DoRothEA) and activity method (Consensus, VIPER, etc.). Our results suggest that cancer type does indeed explain 51% of the variance in the model whilst regulon source and activity estimation method explain much less of the variance: 21% and <0.01%, respectively (Supplementary Figure S1). Thus, cancer type has a bigger influence than the regulon source, and the activity estimation method shows no consistent contribution.

It is reasonable to ask why cancer type has such a big influence—why would AML cell lines have higher average correlations between activity and gene sensitivity than HNSC cell lines? We reasoned that there might be two explanations: (i) the different numbers of cell lines for each cancer type (ranging from 24 to 51) may result in different distributions of correlations and (ii) there may be more transcriptomic diversity in the cell lines from different cancer types. The latter might occur if the cancer type in question has more intrinsic heterogeneity or simply if the cell lines available cover more diverse subtypes. We found that there is a strong correlation between $\overline {| R |}$ and the number of cell lines used for our analysis (Pearson's *R* = −0.89, *P* < 0.01). In fact, adding the number of cell lines as a variable in the linear model, shows that it explains 29% of the variance when cancer type is excluded, but does not explain any additional variance to cancer type. We find that variance in mRNA abundance together with number of cell lines explained 44% of the variance in the linear model, which is 86% of the variance explained by cancer type (Supplementary Figure S1). This suggests the majority of the variance explained by cancer type is in fact explained by the number of cell lines analyzed and the variance in mRNA abundance of these cell lines, with unknown factors contributing ∼7% in the model.

Regulons vary greatly in the number of targets they regulate: from 10 to 386 in DoRothEA regulons and between 1 and 2,103 in GRNdb regulons. Additionally, a target gene can be regulated by multiple regulons. These factors may affect the accuracy with which a regulatory gene's activity is calculated due to the increased complexity of a regulon. To address this problem, we stratified our analysis based on the regulon size and on the number of unique genes regulated by each regulatory gene. Our results suggest that the size of the regulons tested or the number of targets they regulate do not seem to be associated with higher or lower correlations with gene essentiality using either the GRNdb or DoRothEA regulons (Supplementary Figures S2 and S3). Including regulon size and number of unique targets in our linear model analysis shows that both variables explain very little variance compared to the other factors previously identified. Regulon size explains 6% of the variance and the percentage of unique targets that a regulon regulates does not explain any of variance in the model (Supplementary Figure S4A, B).

### Regulons convey cancer type-specific information in relation to gene sensitivity to inhibition

As noted, GRNs for ARACNe and GRNdb are cancer type-specific. We wished to assess whether cancer type-matched GRNs were more informative for predicting gene sensitivity than cancer type-mismatched GRNs. For each cancer type, we ran decoupleR with the cancer type-matched regulons as well as with the nine regulons from the other cancer types (Figure 1A).

**Figure 3.**
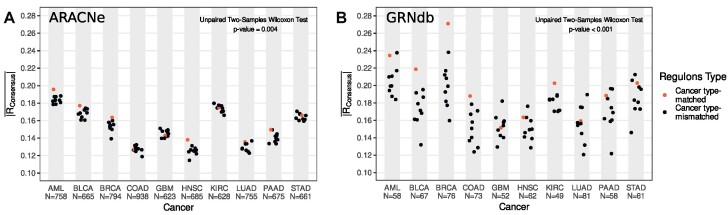
Cancer type-matched regulons predict sensitivity to inhibition better than mismatched regulons. (**A, B**) Absolute Pearson correlation between consensus activity and sensitivity for each cancer paired with every regulon. Each dot represents the average absolute Pearson's correlation coefficients between regulatory gene activity and gene sensitivity across all regulatory genes. (**A**) ARACNe. (**B**) GRNdb (*N* = number of regulatory genes used to generate $\overline {| R |}$ for each cancer type).

Our results suggest that, on average, for all regulon sources and activity estimation methods, except for MLM, cancer type-matched regulons result in a higher absolute correlation between activity and sensitivity than cancer type-mismatched ones (unpaired two-samples Wilcoxon test *P*-value < 0.01) (Figure 3A, B, Supplementary Table S1). Although cancer type-matched regulons were inferred from patient samples and tested in cell lines, our results suggest that tissue-specific regulon interactions are more relevant, as previously suggested (21), thereby improving inference of regulatory gene activity and correlation with gene sensitivity in the DepMap. However, despite cancer type-matched regulons performing better than cancer type-mismatched ones, the correlation between activity and gene sensitivity is still relatively poor on average ($\overline {| R |}$ < 0.28).

### Gene sensitivity to inhibition is better predicted by mRNA abundance than by GRN-inferred activity

We have so far discussed the correlation between regulatory gene activity and gene sensitivity. We have slightly touched on the simpler approach of just using mRNA abundances to predict sensitivity to inhibition. Such a comparison is important for understanding whether the activity estimation methods provide an advantage for predicting gene sensitivity over plain transcript abundance.

Visual inspection of Figure 2B–D suggests that mRNA abundance has a higher correlation with gene sensitivity to inhibition than any of the gene activity estimation methods. This is true across all cancer types analyzed, across all activity estimation methods, and across all GRN sources (Figure 2B–D). While direct comparison of the correlations between regulons from different sources (e.g. ARACNe versus DoRothEA) is challenging due to coverage of different gene sets by each regulon source, this is not the case when comparing the activity estimation methods to mRNA abundance. When comparing the average correlation of activity estimation methods from ARACNe regulons to mRNA abundance we did so over the same set of genes.

We compared the $\overline {| R |}$ derived from mRNA abundance with that derived from Consensus (the best performing individual activity estimation method) across all regulon sources and all cancer types. We found that mRNA abundance had a significantly higher $\overline {| R |}$ (Wilcoxon paired test *P*-value = 6.9 × 10^−6^). Overall, this suggests that the average correlation between mRNA abundance and sensitivity to inhibition is higher than that for any of the inferred activity methods using any of the GRNs.

**Figure 4.**
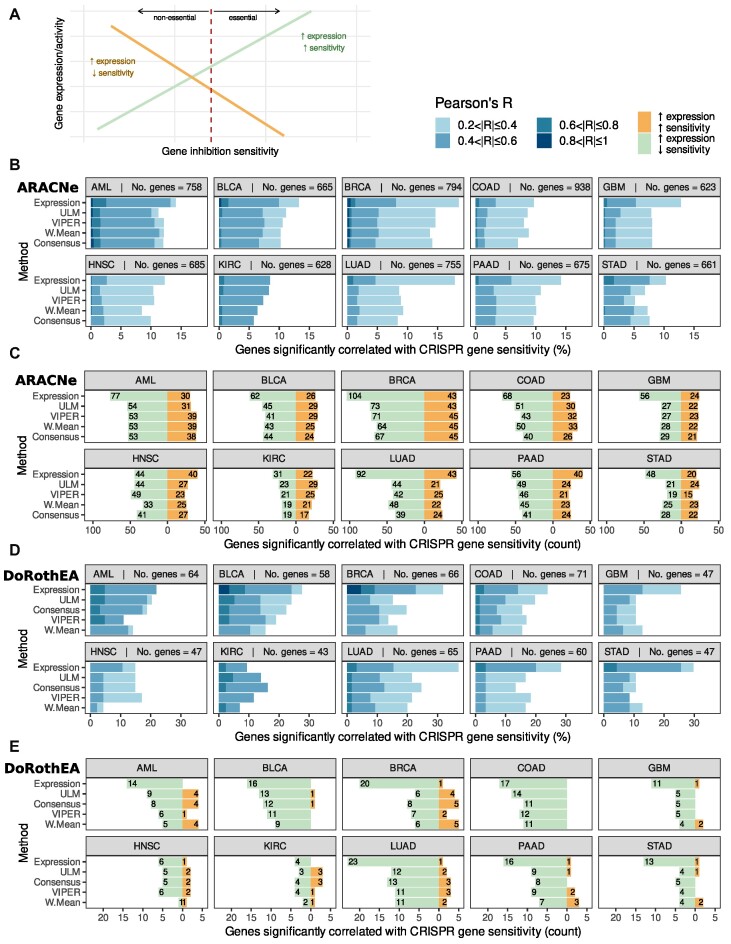
Gene sensitivity to inhibition correlates better with expression than with inferred activity. (**A**) Distinction between the two types of correlations between expression/activity and gene sensitivity score. An increase in expression can be correlated with higher sensitivity (in green). An increase in expression/activity can also be associated with a decrease in sensitivity (in orange). (**B, D**) Pearson's correlation coefficients between activity/expression and gene sensitivity stratified incrementally from |*R*| = 0.2 to 1 to show the percentage of significant regulatory genes correlated with gene sensitivity (*P* < 0.05) after filtering out genes that are never essential and genes that are always essential in a cancer. Methods are sorted top-to-bottom in order of performance across all cancer types for the GRN-inferred method in cause. (**B**) ARACNe regulons. (**D**) DoRothEA regulons. (**C, E**) Analysis of the positive and negative correlations between activity/expression and gene sensitivity shows there are more negative correlations, suggesting there are more cases where an increase in sensitivity is associated with increased expression/activity. Analysis also confirms expression is better correlated with gene sensitivity. (**C**) ARACNe regulons. (**E**) DoRothEA regulons.

However, for the purpose of identifying new therapeutic targets, strong correlations are more important—those that are highly predictive of gene sensitivity. We therefore compared the proportion of genes that show significant correlations between sensitivity and activity to those with significant correlations between sensitivity and mRNA abundance. We found that strong correlations with gene sensitivity (*P* < 0.05, |*R*| > 0.2) were rare. Across all cancer types, neither expression, nor activity had strong correlations with >20% of genes (Figure 4B, D, Supplementary Figure S5A). For nine out of ten cancer types we studied, more genes had a strong correlation between their mRNA abundance and sensitivity than their activity and sensitivity (Figure 4B, D). KIRC, which has the fewest number of cell lines, was the only cancer type where inferred activity was comparable. Expression consistently had more high correlations than activity across all GRN sources and all activity estimation methods (Figure 4B, D, Supplementary Figure S5A). The same trend is evident if the threshold for strong correlations is set at (*P* < 0.05, $\overline {| R |}$ > 0.4) or (*P* < 0.05, $\overline {| R |}$ > 0.6). This suggests that, regardless of the exact threshold used to define a strong correlation, gene expression displays more strong correlations with gene sensitivity. Neither GRNdb-inferred activity, nor DoRothEA-inferred activity perform better than mRNA abundance, confirming the findings of ARACNe-inferred activity (Figure 4D, Supplementary Figure S5A). Additionally, we looked at genes with |R| > 0.8 and across all cancer types. At this threshold we discovered two genes whose sensitivity to inhibition is correlated with inferred activity, but not by mRNA abundance: *FOXA1* by both the ARACNe and GRNdb regulons in BRCA and *KLF1* by ARACNe regulons in AML. However, when we investigate the correlations between the expression of these genes and their sensitivity to inhibition, whilst the threshold of |*R*| > 0.8 is not reached, we see that their correlation is still extremely high (|*R*| > 0.7 in all cases) (Supplementary Table S2, Supplementary Figure S6B, C). Rather than being a radically distinct predictor, this suggests that the activity estimation methods are only slightly better in these few instances.

However, we wished to know whether the genes found as significant by inferred activity and plain expression were the same or different across the different regulon sources. We investigated the overlaps for genes with |*R*| > 0.4 and genes with |*R*| > 0.6 for BRCA and COAD. We note that there are no genes that are common to the three GRN methods but not identified by expression at *R* > 0.4 and only one gene at *R* > 0.6 (Supplementary Figure S5C-F). Additionally, there are very few regulatory genes which overlap in any two of the three methods and are not also identified by expression.

Whilst expression correlated better with sensitivity to inhibition on average, there were specific cases where inferred activity was a better predictor of sensitivity to inhibition. For example, *CDX2* activity performs better in COAD: *R*_Consensus_ = −0.68, *R*_Expression_ = −0.49 (Supplementary Table S2). However, we did not find any consistent pattern that explained why these genes were exceptions. For example, GO enrichment analysis did not reveal any specific functional enrichment in genes for which their sensitivity to inhibition is better correlated with activity than expression. Additionally, we found no enrichment for oncogenes among these cases, according to the CGC (34), or for master regulators, as listed by Paull *et al.* (35). Additionally, we have performed GO enrichment analysis on genes with an absolute correlation between Consensus activity/expression and sensitivity to inhibition >0.6 (Supplementary Table S3). We find the largest number of GO terms enriched when analyzing genes strongly correlated with gene expression. We note that there are no GO terms enriched for genes found using DoRothEA regulons. For the GRN reconstruction methods, only one unique term is enriched among genes inferred using ARACNe regulons and eight terms are inferred using GRNdb regulons. No enriched terms are common across the two GRN methods (Supplementary Figure S6A).

To investigate the correlation between activity/expression and sensitivity to inhibition independently of cancer type, we performed the same analysis at a pan-cancer level, using all cell lines (*n* = 973). We calculated activity based on DoRothEA regulons, as they are cancer type-agnostic (Supplementary Figure S7A, C, E). We found a similar trend: ∼50% of sometimes-essential genes having a correlation >0.2 between mRNA abundance and sensitivity to inhibition (Supplementary Figure S7A). The activity inference methods have strong correlations with fewer genes (25/90 genes with |*R*| > 0.2 for Consensus versus 43/90 for mRNA abundance). Additionally, expression found two extremely high correlations (|*R*| > 0.8), whilst activity found none.

One potential explanation for gene expression having higher absolute correlations with gene sensitivity could be that expression measurements display higher variance than activity scores. However, comparing the per gene variance across our methods shows that gene activities, as determined by VIPER and ULM, have a comparable variance to expression, while activities determined by W. Sum have a significantly higher variance. W. Mean, MLM and Consensus have a slightly lower variance than expression (Supplementary Figure S8).

One limitation of testing the performance of regulons derived from patient samples is that these regulons may not be representative for tumor cells only as patient samples contain a mix of tumor and non-tumor cells. Genes expressed in other cell types, but not in the tumor cells, may be a confounder for the regulon inference. To assess the impact of this, we have performed the same analysis using regulons inferred only from tumor cell lines. To build the regulons, we used the expression matrix containing all cancer cell lines of a cancer type (i.e. not just the cell lines that have both gene expression and gene essentiality in DepMap). We built ARACNe and GRNdb-like regulons (see Methods) for BRCA, COAD, LUAD and PAAD, as they are the cancer types with the largest number of cell lines (excluding HNSC, which is highly heterogeneous due to the large number of subtypes it encompasses). Our analysis suggests that regulons inferred from the CCLE are still not suited for the task of detecting molecular vulnerabilities when paired with activity inference methods. Using ARACNe-inferred regulons we still saw that gene inhibition sensitivity was better correlated with gene expression than with any activity estimation method for BRCA, LUAD and PAAD (Supplementary Figure S8A). For GRNdb-like regulons we noticed ULM and Consensus performing slightly better than expression for BRCA and COAD, but not for LUAD and PAAD (Supplementary Figure S8B). Additionally, using ARACNe regulons there is a larger number of high correlations (|*R*| > 0.6) between sensitivity to inhibition and expression than with any of the activity methods for all four cancer types (Supplementary Figure S8C). For BRCA GRNdb-like regulons, we saw Consensus identifying two very high correlations (>0.8): *CTNNB1* and *FOXA1*, whilst Expression finds *GATA3* with a correlation >0.8 (Supplementary Figure S8E). Taken together, these results suggest that even with GRNs inferred from tumor cell lines only, the performance of activity-estimation methods is not notably better than simply using mRNA abundance.

### Increased sensitivity to gene inhibition is more commonly correlated with increased expression, rather than decreased expression

Thus far, we have focused on the analysis of absolute correlations between activity/expression and sensitivity to inhibition. As noted previously, this is because we anticipated there may be two distinct effect types associated with different genes—sometimes increased expression/activity may be associated with increased sensitivity to inhibition, as observed for oncogene addiction effects, while in other cases reduced expression/activity may be associated with increased sensitivity (Figure 4A). We sought to understand which type of effect was more common, and whether there were differences between inferred activity and gene expression. We found that for gene expression there were consistently more genes where higher expression was associated with increased gene inhibition sensitivity, as previously suggested by other studies (Figure 4C, E, Supplementary Figure S5B) (39,40). This strong skew towards increased expression – increased sensitivity correlations was less evident for the activity methods, e.g. in AML, using ARACNe regulons, 58% of significant genes (53/91) showed an increase in sensitivity with increased Consensus activity. Across the same gene set 72% of significant genes (77/107) showed an increase in sensitivity with increased gene expression.

There was some variation across the different GRN inference methods, ARACNe in general was associated with a much lower proportion of increased activity – increased sensitivity correlations than GRNdb (Figure 4C, Supplementary Figure S5B). However, across both regulon sources, increased expression was consistently associated with an increase in sensitivity.

Interestingly, the use of curated regulons from DoRothEA led to very few cases where an increase in expression/activity results in a decreased sensitivity (Figure 4E, Supplementary Figure S7B, D, F). This suggests that the TFs included in DoRothEA are skewed towards those for which increased activity/expression is associated with increased inhibition sensitivity.

### Expression better predicts binary essentiality

So far, we have analyzed gene inhibition sensitivity from CRISPR screens as a quantitative trait. However, in many cases the results of CRISPR screens are binarized, such that genes are deemed to be either essential or non-essential for survival (39,41,42). Genes which are essential in a specific context might then be considered as suitable therapeutic targets.

To assess the ability of gene activity and gene expression to predict binary essentiality, in each cell line we separated genes into two groups: essential and non-essential (see Methods) (Figure 1B). We then compared the ability of expression and inferred activity to separate the two groups using a Wilcoxon test and the CLES. The interpretation of the CLES is equivalent to the area under the receiver operating characteristic curve (AUC ROC) often used to evaluate binary classifiers. The CLES represents the probability that a gene sampled at random from the essential group will have a higher gene activity/expression than a gene sampled at random from the non-essential group (43). We consider that a gene's essentiality can be well predicted by expression/activity if CLES >0.7 and *P*-value <0.05.

**Figure 5.**
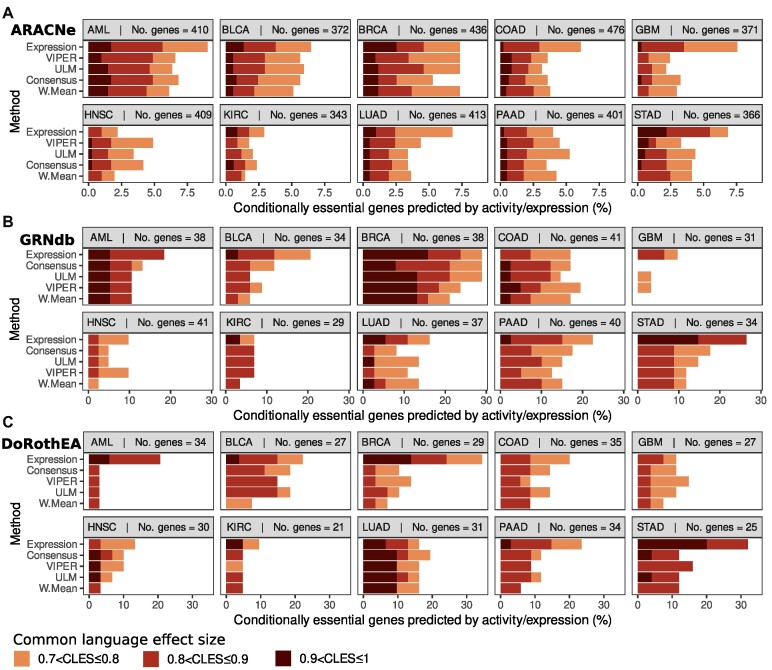
Gene essentiality correlates better with expression than with inferred activity (**A–C**) CLES between activity/expression and binary gene essentiality stratified incrementally from CLES = 0.7 to 1 to show the percentage of significant conditionally essential genes predicted by activity/expression (*P* < 0.05) after filtering out genes that are not essential and genes that are essential in less than three cell lines in a cancer type. Methods are sorted top-to-bottom in order of performance across all cancer types for the GRN-inferred method in cause. (**A**) ARACNe regulons. (**B**) GRNdb regulons. (**C**) DoRothEA regulons.

Our results suggest that, on average, gene expression better predicts binary essentiality, irrespective of whether ARACNe, GRNdb or DoRothEA regulons were used (Figure 5A–C, Supplementary Table S4). In 20 of 30 cases across all regulon types (ten cancer types × three regulon sources), more genes have a CLES >0.7 when their essentiality is predicted using expression, rather than activity. The same is true for different thresholds – for CLES >0.8 and >0.9, expression still predicts more essential genes overall than any of the activity estimation methods using any of the regulon sources. Similarly, on the DoRothEA pan-cancer analysis we found that expression finds ∼51% of sometimes-essential genes with a CLES >0.7 and ∼32% of genes with a CLES >0.9. Consensus, the best performing activity method finds ∼28% genes with a CLES >0.7 (Supplementary Figure S9). Thus, gene expression, rather than inferred activity, is a better predictor of binary gene essentiality.

## Discussion

Our systematic analysis suggests that gene expression performs better than GRN-inferred activity at predicting sensitivity to CRISPR gene inhibition in cancer. This is true regardless of the GRN source and activity inference method used and whether essentiality is treated as a binary or quantitative trait. Whilst extensively used to find ‘master regulators’ of cancer (30), regulatory gene activity does not outperform gene expression for the task of predicting gene sensitivity to inhibition. Across ten cancer types and at a pan-cancer level, more genes are found to have a strong correlation between sensitivity and mRNA abundance than they do between sensitivity and inferred activity.

We find that matched regulons may be more accurate in describing the regulatory gene activity landscape of each cancer type than mismatched regulons. This suggests that there is value to using co-expression information from relevant patient tumors to build GRNs. Additionally, this suggests a degree of similarity between primary patient tumors and cell-line models that can be captured by GRNs. Our study also suggests that cancer type contributes more to the variance in average correlation with gene sensitivity than the GRN-building method or the activity-inference method. This may be at least partially attributable to different cancer types having more variable transcriptomes. We find that there is no significant difference between activity and expression in finding correlations where an increase in sensitivity is associated with decreased expression/activity. However, in all cancer types, there are more genes where an increase in expression, rather than activity, is associated with an increase in sensitivity, in an oncogene addiction-like effect. This is expected to be the case in most cancer cells reliant on the activity of a TF for survival.

Both inferred and curated networks (30) have been used to infer the activity of TFs and to investigate the differential activity of TFs in different conditions (21,44). The assumption behind these approaches is that the activity of a TF inferred from the expression profile of its targets can help uncover hidden potential therapeutic targets. Therefore, these methods have been used as hypothesis-creation tools to find novel targets (21) or associations with patient survival (45). A significant limitation of TF activity estimation approaches is that typically only a small number of candidate targets are selected for experimental testing (17). It is thus extremely challenging to understand how broadly useful these approaches are, and to estimate false positive or false negative rates.

We propose a computational approach based on CRISPR screen data to assess the ability of GRN-inferred activity to predict sensitivity to perturbation in tumor cell lines. The repository of cell lines being screened grows every year, offering more statistical power (2–5). A significant advantage of our approach is that it is unbiased, in the sense that all genes are evaluated, rather than one or two selected candidates. Evaluating only one or two candidates may lead to a false sense of accuracy of the approach, downplaying its limitations. A limitation of our approach is that we are evaluating a downstream use of inferred GRNs rather than the GRNs themselves, i.e. we have not evaluated the ability of the reconstructed networks to predict transcriptional changes, but rather their ability to predict therapeutic targets. However, the latter is a purpose for which they are often employed.

Our study is primarily limited by the availability of the data, as there is a limited number of cell lines with genome-wide CRISPR screens data. We tried to mitigate this by selecting cancer types with a large number of cell lines screened. However, we see the number of cell lines used contributes 29% to the variance explained by the model used to predict the average absolute Pearson's correlation between activity and sensitivity to inhibition, suggesting the results from cancer types with a low number of cell lines available (i.e. STAD, AML) may be less reliable in our analysis. Furthermore, the gene sensitivity measurements we use are made in cell line models. These might not entirely reflect cancer cells within actual tumors, surrounded by the tumor microenvironment. Additionally, our results are reflective of cohorts of cell lines displaying a range of regulatory landscapes. A better approach might be to integrate multiple sources of data for model-specific GRNs, as done by Goode *et al.* (46) and Assi *et al.* (47). They create GRNs from multiple omics sources that are cell line-specific.

We have assessed the ability of GRNs to predict gene essentiality as measured in CRISPR screens. Other methodologies, such as RNA interference (RNAi), have also been used to assess gene essentiality in large numbers of cell lines (48,49). While in general CRISPR screens appear better at identifying essential genes than RNAi-based methods (50,51), there is also evidence that combining CRISPR-based gene essentiality scores with RNAi-based scores can be especially informative for understanding variation in pan-essential genes (52,53). Therefore, there may be value in assessing the ability of GRNs to predict gene essentiality derived from RNAi.

Ultimately, our study primarily looks at correlations between GRN-inferred activity and sensitivity and future work could explore indirect relationships between the GRNs and sensitivity to inhibition. For instance, Garcia-Alonso *et al.* (21) explores the relationships between drug sensitivity and the activity of indirect targets, finding correlations with clinical significance. However, despite these caveats, expression consistently outperforms GRN-inferred activity in predicting sensitivity to CRISPR inhibition in a variety of cancer types. This work underlines the utility of sensitivity data from CRISPR screens in benchmarking the use of GRN-inferred activity methods for nominating therapeutic targets.

## Supplementary Material

zcad056_Supplemental_Files

## Data Availability

R code to run analyses is available at https://github.com/cancergenetics/GRN_activity_corr_essentiality and https://doi.org/10.5281/zenodo.8256519. Correlations between gene essentiality and inferred activity/expression are available on figshare via DOI: 10.6084/m9.figshare.23858484.

## References

[1] Ghandi M., Huang F.W., Jané-Valbuena J., Kryukov G.V., Lo C.C., McDonald E.R., Barretina J., Gelfand E.T., Bielski C.M., Li H. et al. Next-generation characterization of the Cancer Cell Line Encyclopedia. Nature. 2019; 569:503–508. 31068700 10.1038/s41586-019-1186-3 PMC6697103

[2] Dempster J.M., Boyle I., Vazquez F., Root D.E., Boehm J.S., Hahn W.C., Tsherniak A., McFarland J.M. Chronos: a cell population dynamics model of CRISPR experiments that improves inference of gene fitness effects. Genome Biol. 2021; 22:343. 34930405 10.1186/s13059-021-02540-7 PMC8686573

[3] Dempster J.M., Rossen J., Kazachkova M., Pan J., Kugener G., Root D.E., Tsherniak A. Extracting biological insights from the project Achilles genome-scale CRISPR screens in cancer cell lines. 2019; bioRxiv doi:31 July 2019, preprint: not peer reviewed 10.1101/720243.

[4] Meyers R.M., Bryan J.G., McFarland J.M., Weir B.A., Sizemore A.E., Xu H., Dharia N.V., Montgomery P.G., Cowley G.S., Pantel S. et al. Computational correction of copy number effect improves specificity of CRISPR-Cas9 essentiality screens in cancer cells. Nat. Genet. 2017; 49:1779–1784. 29083409 10.1038/ng.3984 PMC5709193

[5] Pacini C., Dempster J.M., Boyle I., Gonçalves E., Najgebauer H., Karakoc E., van der Meer D., Barthorpe A., Lightfoot H., Jaaks P. et al. Integrated cross-study datasets of genetic dependencies in cancer. Nat. Commun. 2021; 12:1661. 33712601 10.1038/s41467-021-21898-7 PMC7955067

[6] Garnett M.J., Edelman E.J., Heidorn S.J., Greenman C.D., Dastur A., Lau K.W., Greninger P., Thompson I.R., Luo X., Soares J. et al. Systematic identification of genomic markers of drug sensitivity in cancer cells. Nature. 2012; 483:570–575. 22460902 10.1038/nature11005 PMC3349233

[7] Iorio F., Knijnenburg T.A., Vis D.J., Bignell G.R., Menden M.P., Schubert M., Aben N., Gonçalves E., Barthorpe S., Lightfoot H. et al. A landscape of pharmacogenomic interactions in cancer. Cell. 2016; 166:740–754. 27397505 10.1016/j.cell.2016.06.017 PMC4967469

[8] Bushweller J.H. Targeting transcription factors in cancer — from undruggable to reality. Nat. Rev. Cancer. 2019; 19:611–624. 31511663 10.1038/s41568-019-0196-7 PMC8820243

[9] Alessandrini F., Pezzè L., Menendez D., Resnick M.A., Ciribilli Y. ETV7-mediated DNAJC15 repression leads to doxorubicin resistance in breast cancer cells. Neoplasia. 2018; 20:857–870. 30025229 10.1016/j.neo.2018.06.008 PMC6077117

[10] Ohanian M., Rozovski U., Kanagal-Shamanna R., Abruzzo L.V., Loghavi S., Kadia T., Futreal A., Bhalla K., Zuo Z., Huh Y.O. et al. MYC protein expression is an important prognostic factor in acute myeloid leukemia. Leuk. Lymphoma. 2019; 60:37–48. 29741984 10.1080/10428194.2018.1464158 PMC6226369

[11] Henley M.J., Koehler A.N. Advances in targeting ‘undruggable’ transcription factors with small molecules. Nat. Rev. Drug Discov. 2021; 20:669–688. 34006959 10.1038/s41573-021-00199-0

[12] Lefebvre C., Rieckhof G., Califano A. Reverse-engineering human regulatory networks. Wiley Interdiscip. Rev. Syst. Biol. Med. 2012; 4:311–325. 22246697 10.1002/wsbm.1159 PMC4128340

[13] Barabási A.L., Gulbahce N., Loscalzo J. Network medicine: a network-based approach to human disease. Nat. Rev. Genet. 2011; 12:56–68. 21164525 10.1038/nrg2918 PMC3140052

[14] Lachmann A., Giorgi F.M., Lopez G., Califano A. ARACNe-AP: gene network reverse engineering through adaptive partitioning inference of mutual information. Bioinformatics. 2016; 32:2233–2235. 27153652 10.1093/bioinformatics/btw216 PMC4937200

[15] Huynh-Thu V.A., Irrthum A., Wehenkel L., Geurts P. Inferring regulatory networks from expression data using tree-based methods. PLoS One. 2010; 5:e12776. 20927193 10.1371/journal.pone.0012776 PMC2946910

[16] Iglesias-Martinez L.F., De Kegel B., Kolch W. KBoost: a new method to infer gene regulatory networks from gene expression data. Sci. Rep. 2021; 11:15461. 34326402 10.1038/s41598-021-94919-6 PMC8322418

[17] Alvarez M.J., Shen Y., Giorgi F.M., Lachmann A., Ding B.B., Hilda Ye B., Califano A. Functional characterization of somatic mutations in cancer using network-based inference of protein activity. Nat. Genet. 2016; 48:838–847. 27322546 10.1038/ng.3593 PMC5040167

[18] Aibar S., González-Blas C.B., Moerman T., Huynh-Thu V.A., Imrichova H., Hulselmans G., Rambow F., Marine J.C., Geurts P., Aerts J. et al. SCENIC: single-cell regulatory network inference and clustering. Nat. Methods. 2017; 14:1083–1086. 28991892 10.1038/nmeth.4463 PMC5937676

[19] Essaghir A., Toffalini F., Knoops L., Kallin A., van Helden J., Demoulin J.B. Transcription factor regulation can be accurately predicted from the presence of target gene signatures in microarray gene expression data. Nucleic Acids Res. 2010; 38:120. 10.1093/nar/gkq149 PMC2887972 20215436

[20] Gocho Y., Liu J., Hu J., Yang W., Dharia N.V, Zhang J., Shi H., Du G., John A., Lin T.-N. Network-based systems pharmacology reveals heterogeneity in LCK and BCL2 signaling and therapeutic sensitivity of T-cell acute lymphoblastic leukemia. Nat. Cancer. 2021; 2:284–299. 34151288 10.1038/s43018-020-00167-4 PMC8208590

[21] Garcia-Alonso L., Iorio F., Matchan A., Fonseca N., Jaaks P., Peat G., Pignatelli M., Falcone F., Benes C.H., Dunham I. et al. Transcription factor activities enhance markers of drug sensitivity in cancer. Cancer Res. 2018; 78:769–780. 29229604 10.1158/0008-5472.CAN-17-1679 PMC6522379

[22] Shaw T.I., Dong L., Tian L., Qian C., Liu Y., Ju B., High A., Kavdia K., Pagala V.R., Shaner B. et al. Integrative network analysis reveals USP7 haploinsufficiency inhibits E-protein activity in pediatric T-lineage acute lymphoblastic leukemia (T-ALL). Sci. Rep. 2021; 11:5154. 33664368 10.1038/s41598-021-84647-2 PMC7933146

[23] Alvarez M.J., Subramaniam P.S., Tang L.H., Grunn A., Aburi M., Rieckhof G., Komissarova E.V., Hagan E.A., Bodei L., Clemons P.A. et al. A precision oncology approach to the pharmacological targeting of mechanistic dependencies in neuroendocrine tumors. Nat. Genet. 2018; 50:979–989. 29915428 10.1038/s41588-018-0138-4 PMC6421579

[24] Wang K., Saito M., Bisikirska B.C., Alvarez M.J., Lim W.K., Rajbhandari P., Shen Q., Nemenman I., Basso K., Margolin A.A. et al. Genome-wide identification of post-translational modulators of transcription factor activity in human B cells. Nat. Biotechnol. 2009; 27:829–837. 19741643 10.1038/nbt.1563 PMC2753889

[25] Sousa A., Dugourd A., Memon D., Petursson B., Petsalaki E., Saez-Rodriguez J., Beltrao P. Pan-cancer landscape of protein activities identifies drivers of signalling dysregulation and patient survival. Mol. Syst. Biol. 2023; 19:10631. 10.15252/msb.202110631 PMC9996241 36688815

[26] Trescher S., Münchmeyer J., Leser U. Estimating genome-wide regulatory activity from multi-omics data sets using mathematical optimization. BMC Syst. Biol. 2017; 11:41. 28347313 10.1186/s12918-017-0419-z PMC5369021

[27] Bhagwat A.S., Vakoc C.R. Targeting transcription factors in cancer. Trends Cancer. 2015; 1:53–65. 26645049 10.1016/j.trecan.2015.07.001 PMC4669894

[28] Bradner J.E., Hnisz D., Young R.A. Transcriptional addiction in cancer. 2017; 168:629–643. 10.1016/j.cell.2016.12.013 PMC5308559 28187285

[29] Shalem O., Sanjana N.E., Zhang F. High-throughput functional genomics using CRISPR-Cas9. Nat. Rev. Genet. 2015; 16:299–311. 25854182 10.1038/nrg3899 PMC4503232

[30] Garcia-Alonso L., Holland C.H., Ibrahim M.M., Turei D., Saez-Rodriguez J. Benchmark and integration of resources for the estimation of human transcription factor activities. Genome Res. 2019; 29:1363–1375. 31340985 10.1101/gr.240663.118 PMC6673718

[31] Fang L., Li Y., Ma L., Xu Q., Tan F., Chen G. GRNdb: decoding the gene regulatory networks in diverse human and mouse conditions. Nucleic Acids Res. 2021; 49:D97–D103. 33151298 10.1093/nar/gkaa995 PMC7779055

[32] Badia-i-Mompel P., Vélez Santiago J., Braunger J., Geiss C., Dimitrov D., Müller-Dott S., Taus P., Dugourd A., Holland C.H., Ramirez Flores R.O. et al. decoupleR: ensemble of computational methods to infer biological activities from omics data. Bioinforma. Adv. 2022; 2:vbac016. 10.1093/bioadv/vbac016 PMC9710656 36699385

[33] Liao Y., Wang J., Jaehnig E.J., Shi Z., Zhang B. WebGestalt 2019: gene set analysis toolkit with revamped UIs and APIs. Nucleic Acids Res. 2019; 47:W199–W205. 31114916 10.1093/nar/gkz401 PMC6602449

[34] Sondka Z., Bamford S., Cole C.G., Ward S.A., Dunham I., Forbes S.A. The COSMIC Cancer Gene Census: describing genetic dysfunction across all human cancers. Nat. Rev. Cancer. 2018; 18:696–705. 30293088 10.1038/s41568-018-0060-1 PMC6450507

[35] Paull E.O., Aytes A., Jones S.J., Abate-shen C., Alvarez M.J., Califano A. A modular master regulator landscape controls cancer transcriptional identity. Cell. 2021; 184:334–351. 33434495 10.1016/j.cell.2020.11.045 PMC8103356

[36] Ding Y.Y., Kim H., Madden K., Loftus J.P., Chen G.M., Allen D.H., Zhang R., Xu J., Chen C.H., Hu Y. et al. Network analysis reveals synergistic genetic dependencies for rational combination therapy in Philadelphia chromosome-like acute lymphoblastic leukemia. Clin. Cancer Res. 2021; 27:5109–5122. 34210682 10.1158/1078-0432.CCR-21-0553 PMC8448976

[37] Nijhawan D., Zack T.I., Ren Y., Strickland M.R., Lamothe R., Schumacher S.E., Tsherniak A., Besche H.C., Rosenbluh J., Shehata S. et al. Cancer vulnerabilities unveiled by genomic loss. Cell. 2012; 150:842–854. 22901813 10.1016/j.cell.2012.07.023 PMC3429351

[38] Paolella B.R., Gibson W.J., Urbanski L.M., Alberta J.A., Zack T.I., Bandopadhayay P., Nichols C.A., Agarwalla P.K., Brown M.S., Lamothe R. et al. Copy-number and gene dependency analysis reveals partial copy loss of wild-type SF3B1 as a novel cancer vulnerability. Elife. 2017; 6:e23268. 28177281 10.7554/eLife.23268 PMC5357138

[39] Hart T., Brown K.R., Sircoulomb F., Rottapel R., Moffat J. Measuring error rates in genomic perturbation screens: gold standards for human functional genomics. Mol. Syst. Biol. 2014; 10:733. 24987113 10.15252/msb.20145216 PMC4299491

[40] Wang T., Birsoy K., Hughes N.W., Krupczak K.M., Post Y., Wei J.J., Lander E.S., Sabatini D.M. Identification and characterization of essential genes in the human genome. Science. 2015; 350:1096–1101. 26472758 10.1126/science.aac7041 PMC4662922

[41] de Kegel B., Ryan C.J. Paralog buffering contributes to the variable essentiality of genes in cancer cell lines. PLoS Genet. 2019; 15:e1008466. 31652272 10.1371/journal.pgen.1008466 PMC6834290

[42] Vinceti A., Karakoc E., Pacini C., Perron U., De Lucia R.R., Garnett M.J., Iorio F. CoRe: a robustly benchmarked R package for identifying core-fitness genes in genome-wide pooled CRISPR-Cas9 screens. Bmc Genomics (Electronic Resource). 2021; 22:828. 10.1186/s12864-021-08129-5 PMC8597285 34789150

[43] McGraw K.O., Wong S.P. Forming inferences about some intraclass correlation coefficients. Psychol. Methods. 1996; 1:30.

[44] Du X., Wen J., Wang Y., Karmaus P.W.F., Khatamian A., Tan H., Li Y., Guy C., Nguyen T.L.M., Dhungana Y. et al. Hippo/Mst signalling couples metabolic state and immune function of CD8α+ dendritic cells. Nature. 2018; 558:141–145. 29849151 10.1038/s41586-018-0177-0 PMC6292204

[45] Falco M.M., Bleda M., Carbonell-Caballero J., Dopazo J. The pan-cancer pathological regulatory landscape. Sci. Rep. 2016; 6:39709. 28000771 10.1038/srep39709 PMC5175166

[46] Goode D.K., Obier N., Vijayabaskar M.S., Lie-A-Ling M., Lilly A.J., Hannah R., Lichtinger M., Batta K., Florkowska M., Patel R. et al. Dynamic gene regulatory networks drive hematopoietic specification and differentiation. Dev. Cell. 2016; 36:572–587. 26923725 10.1016/j.devcel.2016.01.024 PMC4780867

[47] Assi S.A., Imperato M.R., Coleman D.J.L., Pickin A., Potluri S., Ptasinska A., Chin P.S., Blair H., Cauchy P., James S.R. et al. Subtype-specific regulatory network rewiring in acute myeloid leukemia. Nat. Genet. 2019; 51:151–162. 30420649 10.1038/s41588-018-0270-1 PMC6330064

[48] Tsherniak A., Vazquez F., Montgomery P.G., Weir B.A., Kryukov G., Cowley G.S., Gill S., Harrington W.F., Pantel S., Krill-Burger J.M. et al. Defining a cancer dependency map. Cell. 2017; 170:564–576. 28753430 10.1016/j.cell.2017.06.010 PMC5667678

[49] Campbell J., Ryan C.J., Brough R., Bajrami I., Pemberton H.N., Chong I.Y., Costa-Cabral S., Frankum J., Gulati A., Holme H. et al. Large-scale profiling of kinase dependencies in cancer cell lines. Cell Rep. 2016; 14:2490–2501. 26947069 10.1016/j.celrep.2016.02.023 PMC4802229

[50] Hart T., Chandrashekhar M., Aregger M., Steinhart Z., Brown K.R., MacLeod G., Mis M., Zimmermann M., Fradet-Turcotte A., Sun S. et al. High-resolution CRISPR screens reveal fitness genes and genotype-specific cancer liabilities. Cell. 2015; 163:1515–1526. 26627737 10.1016/j.cell.2015.11.015

[51] Smith I., Greenside P.G., Natoli T., Lahr D.L., Wadden D., Tirosh I., Narayan R., Root D.E., Golub T.R., Subramanian A. et al. Evaluation of RNAi and CRISPR technologies by large-scale gene expression profiling in the connectivity Map. PLoS Biol. 2017; 15:e2003213. 29190685 10.1371/journal.pbio.2003213 PMC5726721

[52] Wang W., Malyutina A., Pessia A., Saarela J., Heckman C.A., Tang J. Combined gene essentiality scoring improves the prediction of cancer dependency maps. EBioMedicine. 2019; 50:67–80. 31732481 10.1016/j.ebiom.2019.10.051 PMC6923492

[53] Krill-Burger J.M., Dempster J.M., Borah A.A., Paolella B.R., Root D.E., Golub T.R., Boehm J.S., Hahn W.C., McFarland J.M., Vazquez F. et al. Partial gene suppression improves identification of cancer vulnerabilities when CRISPR-Cas9 knockout is pan-lethal. Genome Biol. 2023; 24:192. 37612728 10.1186/s13059-023-03020-w PMC10464129

