# Supplementary material for: Gene essentiality in cancer is better predicted by mRNA abundance than by gene regulatory network-inferred activity
Source: NAR Cancer. 2023 Nov 28;5(4):zcad056. doi: 10.1093/narcan/zcad056 (PMC10683780; doi:10.1093/narcan/zcad056)
Supplement: zcad056_Supplemental_Files [file zcad056_supplemental_files.zip › Supplementary_figures_resubmission.pdf]

Supplement

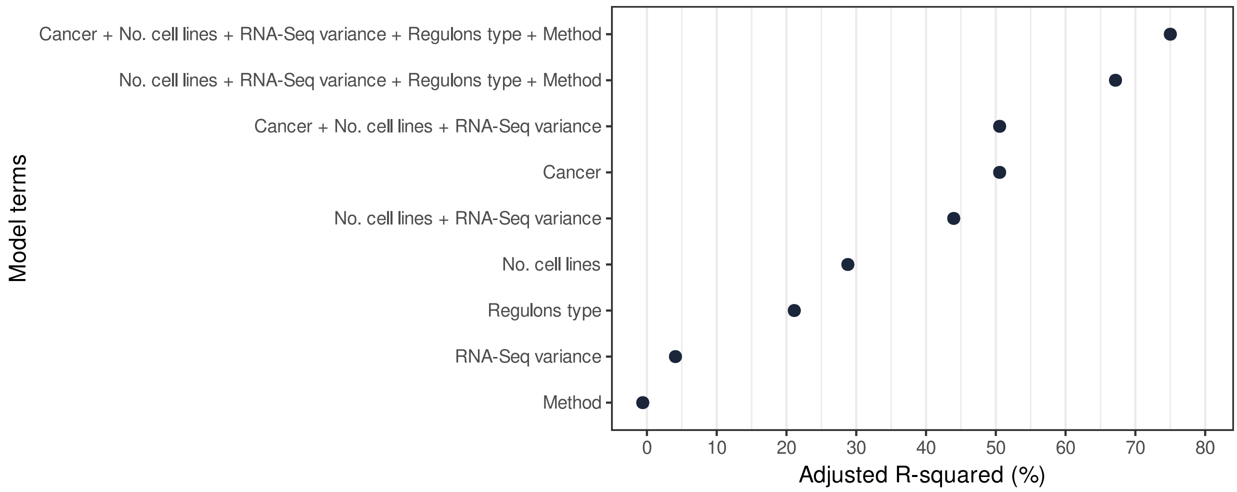

Supplementary Fig. 1 | Each terms' contribution to linear model predicting |R|. Each dot represents the percentage of variance explained (Adjusted R-squared) by each variable in the linear model predicting the absolute correlation between essentiality and activity (|Pearson's R|).

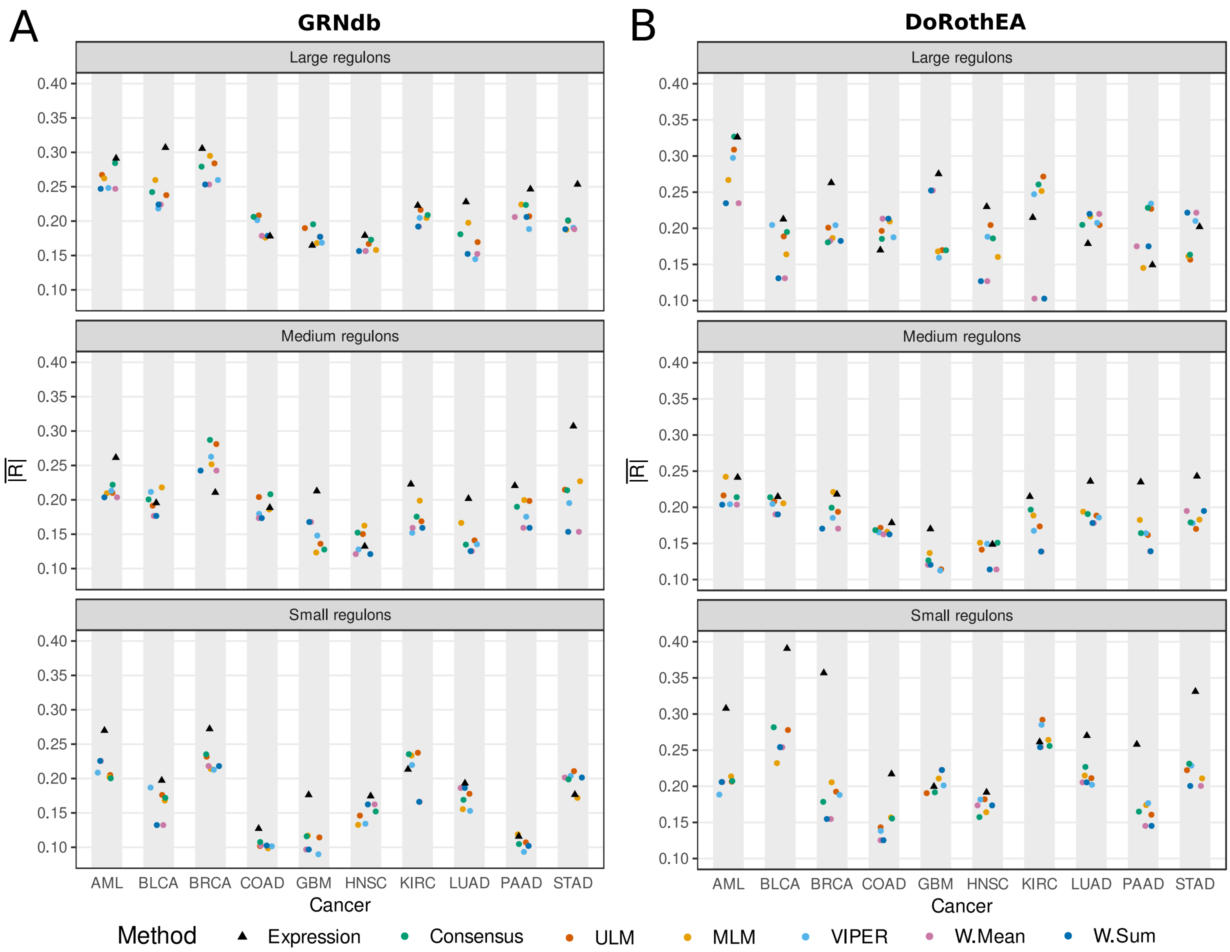

Supplementary Fig. 2 | Differences between activity and expression correlations with gene sensitivity to inhibition are similar independent of regulon size A, B, Comparison between the different inferred activity methods (paired with cancer type-matched regulons) correlating with gene sensitivity and gene expression correlating with gene sensitivity, by regulon size. **A,** ARACNe. **B,** DoRothEA.

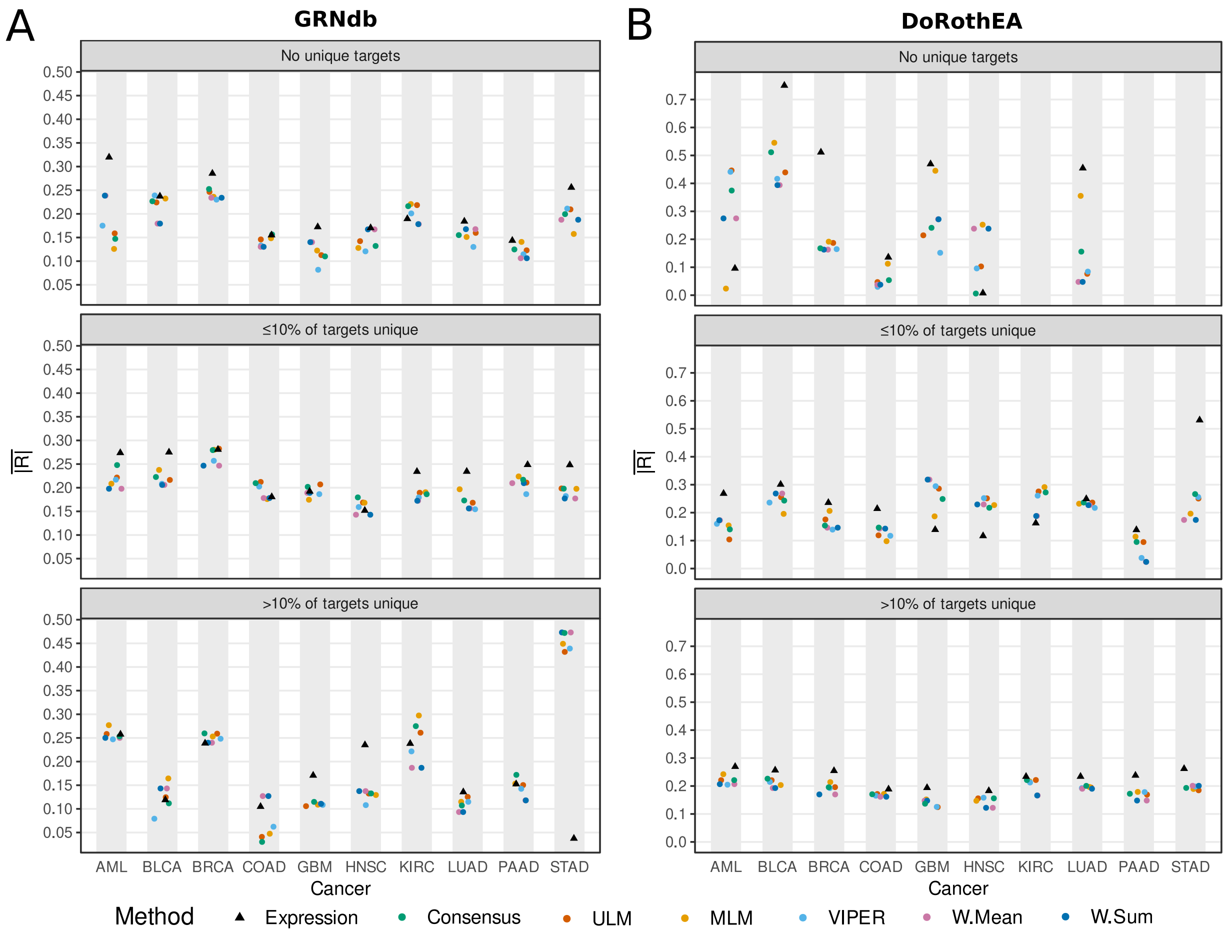

Supplementary Fig. 3 | Differences between activity and expression correlations with gene sensitivity to inhibition are similar independent of the number of unique targets each regulon has A, B, Comparison between the different inferred activity methods (paired with cancer type-matched regulons) correlating with gene sensitivity and gene expression correlating with gene sensitivity, by regulon size. **A,** GRNdb **B,** DoRothEA.

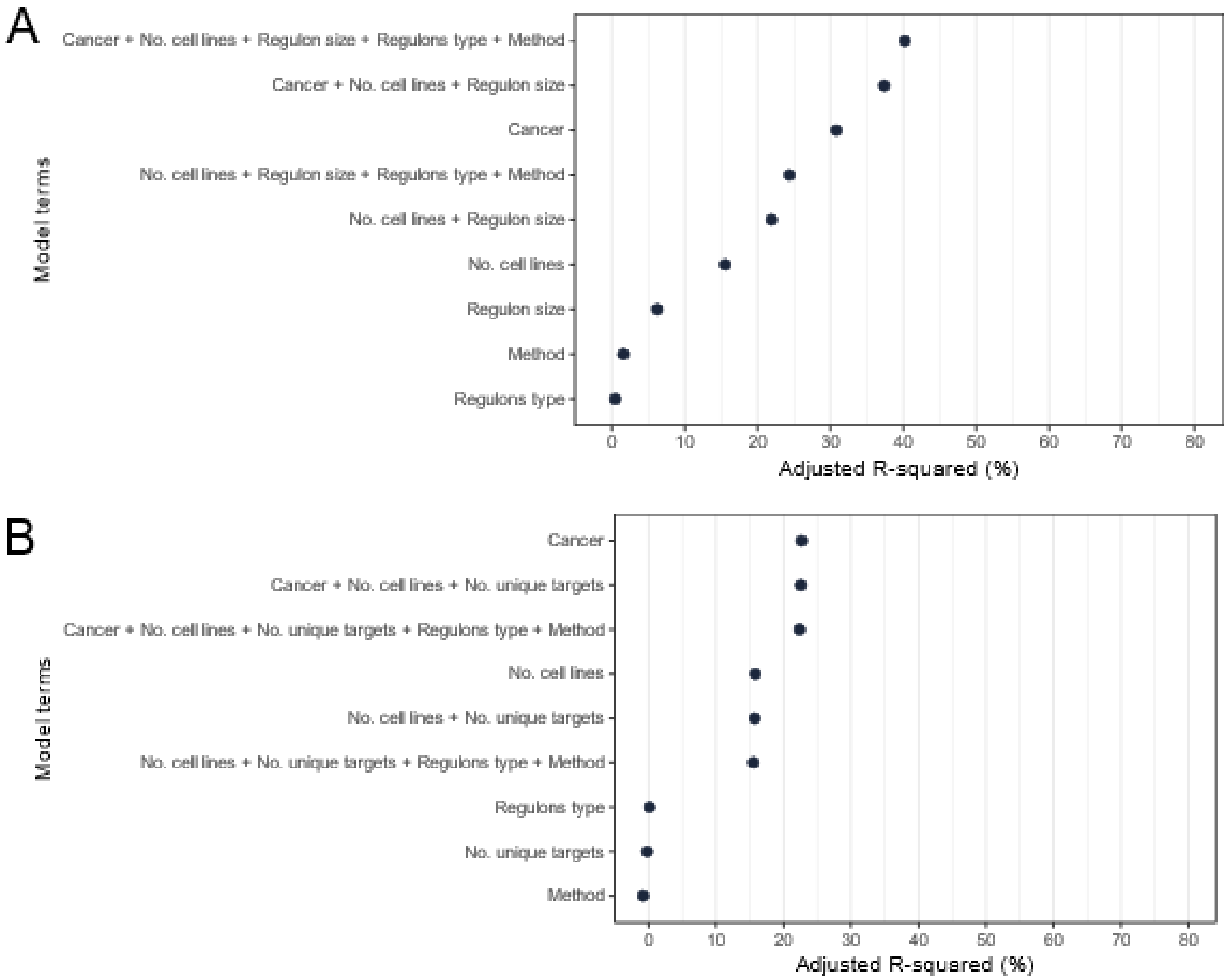

Supplementary Fig. 4 | Each terms' contribution to linear model predicting |R|. A, Using regulon size as to stratify. **B,** Using the number of unique targets each regulon regulates. Each dot represents the percentage of variance explained (Adjusted R-squared) by each variable in the linear model predicting the absolute correlation between essentiality and activity (|Pearson's R|). Note: the percentages are different from Fig 1A, as here the value for |R| is calculated over the stratification variable (i. e., regulon size or number of unique targets) and we only used GRNdb and DoRothEA regulons.

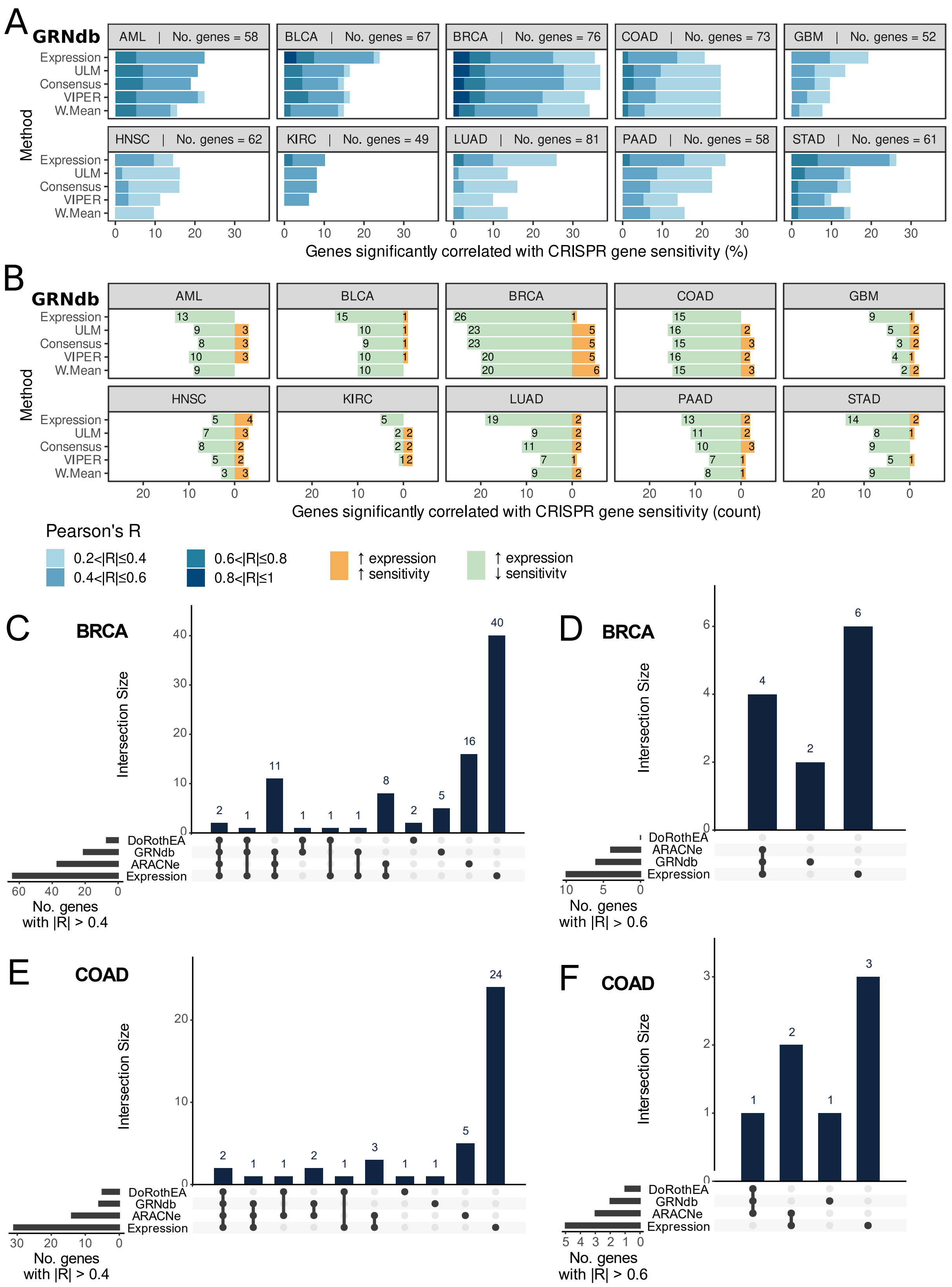

Supplementary Fig. 5 | Gene essentiality correlates better with expression than with inferred activity using GRNdb regulons. A, Pearson's correlation coefficients between activity/expression and gene sensitivity stratified incrementally from |R| = 0.2 to 1 to show the percentage of significant regulatory genes correlated with gene sensitivity ($p < 0.05$) after filtering out genes that are never essential and genes that are always essential in a cancer type. Methods are sorted top-to-bottom in order of performance across all cancer types for the GRN-inferred method in cause. **B,** Analysis of the high expression – high sensitivity and high expression low sensitivity correlations between

activity/expression and gene essentiality shows there are more cases where an increase in sensitivity is associated with increased expression/activity. **C, D,** Overlap of significantly correlated genes between expression/activity and sensitivity to inhibition using GRNdb, ARACNe and DoRothEA regulons in BRCA. C, |R| > 0.4, **D,** |R| > 0.6. **E, F,** Overlap of significantly correlated genes between expression/activity and sensitivity to inhibition using GRNdb, ARACNe and DoRothEA regulons in COAD. **E,** |R| > 0.4, **F,** |R| > 0.6.

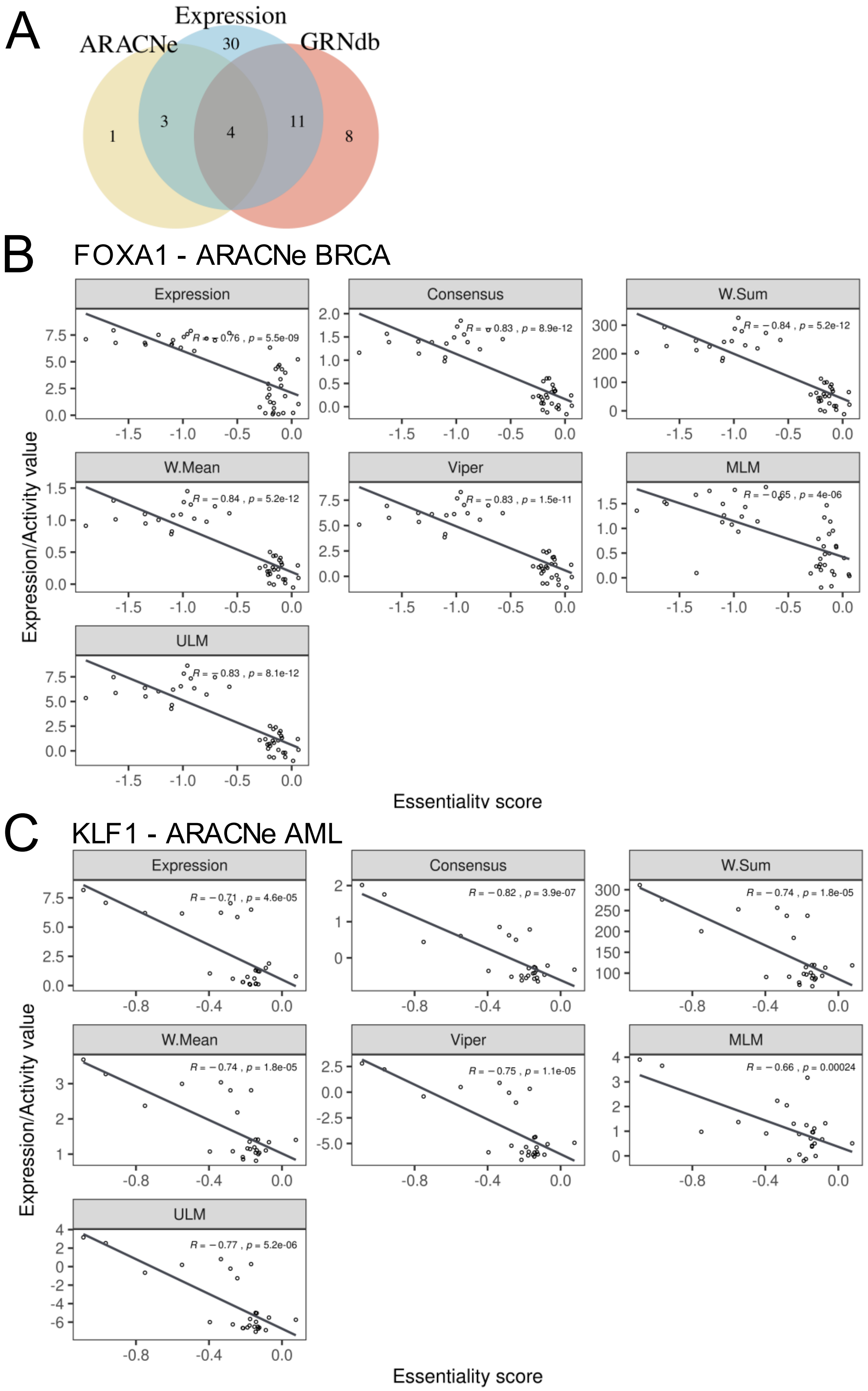

Supplementary Fig. 6. | GO enrichment over GRN methods and individual correlations with |R| > 0.6. A, Overlap between GO terms found as enriched in genes with a correlation > 0.6 between Consensus activity/expression and sensitivity to inhibition. **B, C,** Scatter plots and correlations between

inferred activity/expression for individual genes **B.** *FOXA1* using ARACNe GRNs in BRCA and **C**. *KLF1* using ARACNe GRNs in AML.

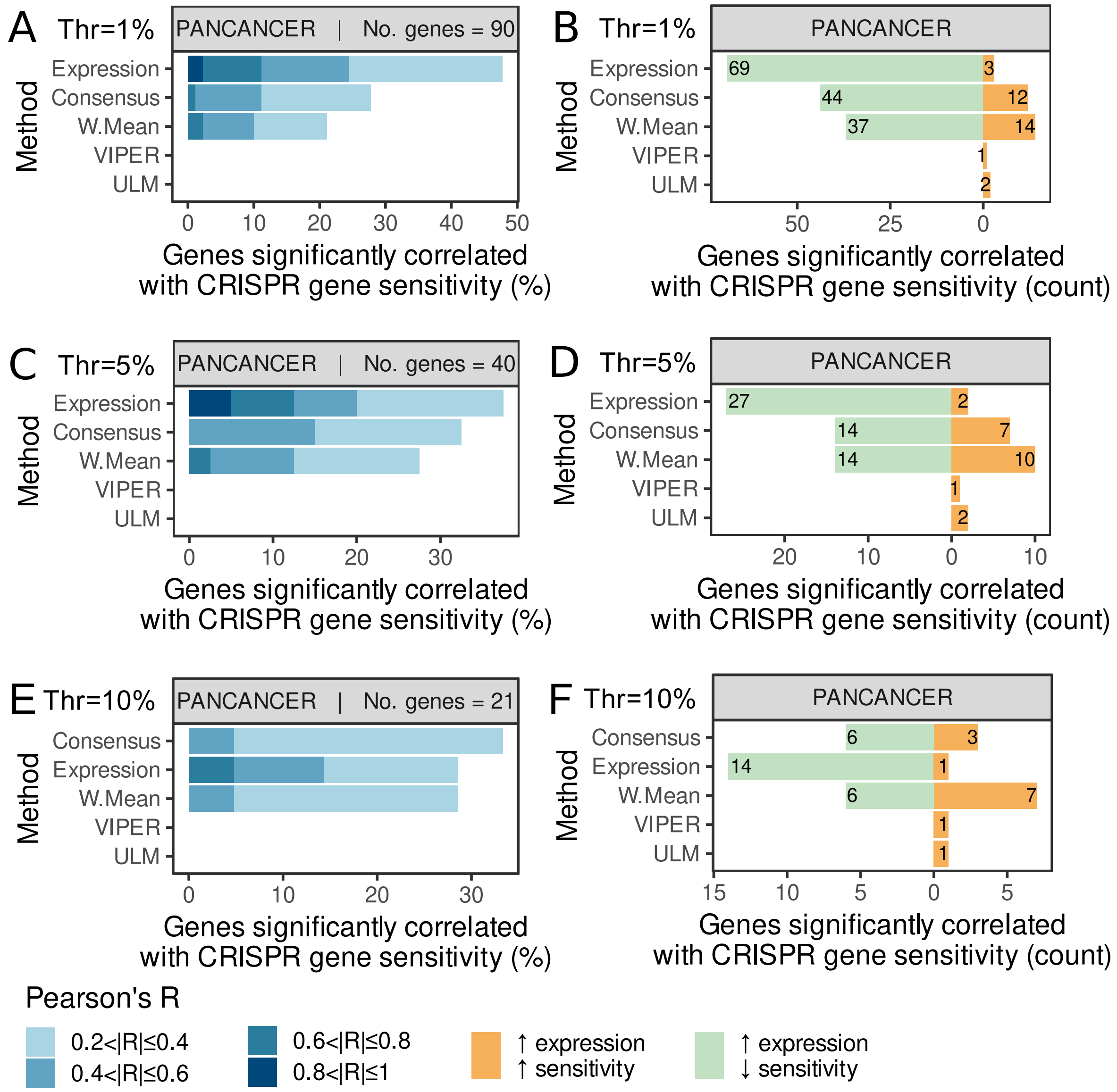

Supplementary Fig. 7 | Gene essentiality correlates better with expression than with inferred activity using literature curated DorothEA regulons in a pancancer analysis regardless of the threshold used to call a gene 'sometimes' essential (see Methods). A, C, E, Pearson's correlation coefficients between activity/expression and gene sensitivity stratified incrementally from |R| = 0.2 to 1 to show the percentage of significant regulatory genes correlated with gene sensitivity ($p < 0.05$) after filtering out genes that are never essential and genes that are always essential in a cancer. Methods are sorted top-to-bottom in order of performance. **A,** Threshold = 1%. **C,** Threshold = 5%. **E,** Threshold = 10%. **B, D, F,** Analysis of the high expression – high sensitivity and high expression low sensitivity correlations between activity/expression and gene essentiality shows there are more cases where an increase in sensitivity is associated with increased expression/activity. **B,** Threshold = 1%. **D,** Threshold = 5%. **F,** Threshold = 10%.

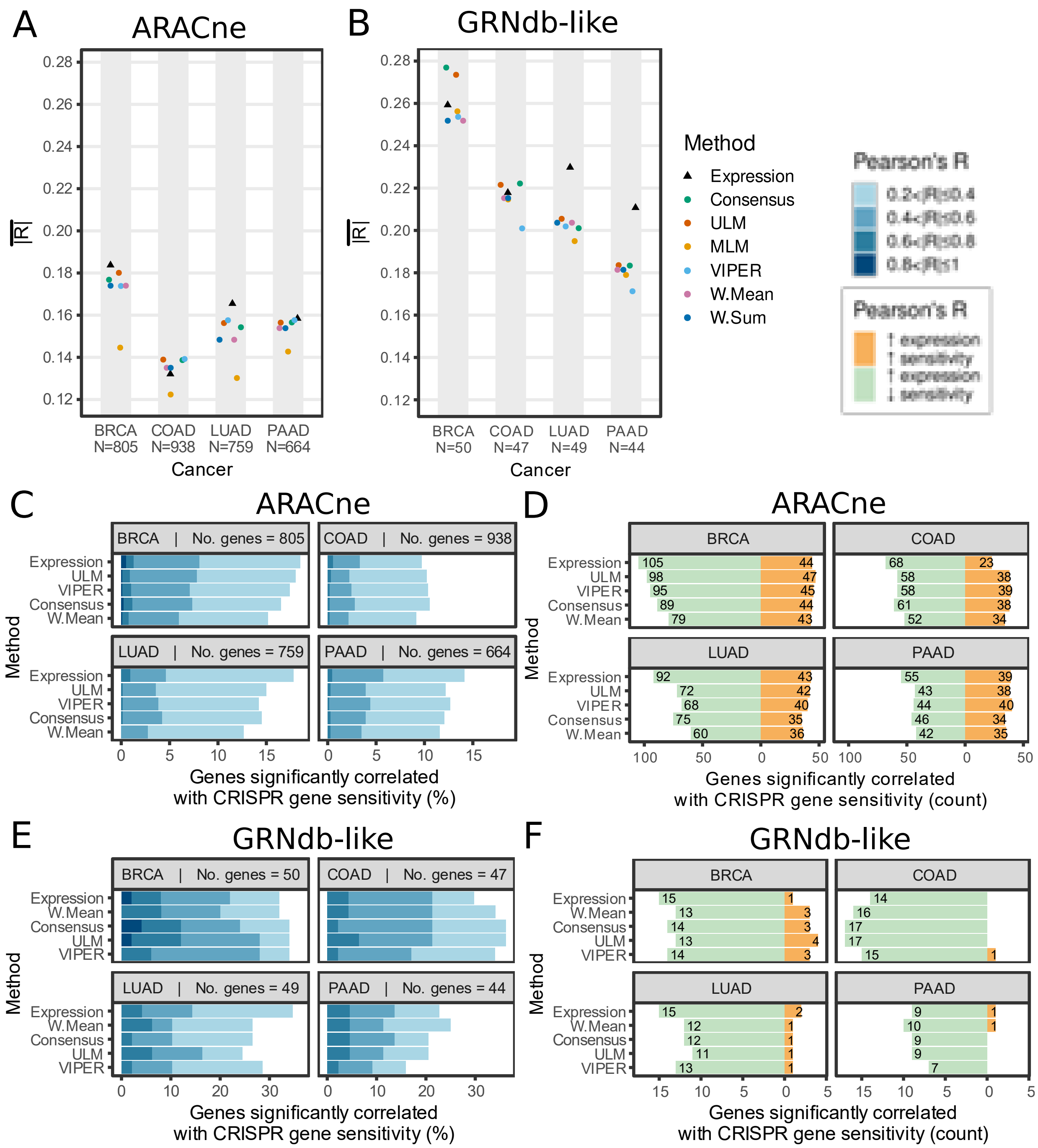

Supplementary Fig. 8 | Correlations between sensitivity to inhibition and GRN-inferred activity/mRNA abundance using regulons inferred from the CCLE. A, B, Comparison between the different inferred activity methods (paired with cancer type-matched regulons) correlating with gene sensitivity and gene expression correlating with gene sensitivity. **C, E,** Pearson's correlation coefficients between activity/expression and gene sensitivity stratified incrementally from |R| = 0.2 to 1 to show the percentage of significant regulatory genes correlated with gene sensitivity ($p < 0.05$) after filtering out genes that are never essential and genes that are always essential in a cancer. Methods are sorted top-to-bottom in order of performance across all cancer types for the GRN-inferred method in cause. **D, F,** Analysis of the high expression – high sensitivity and high expression low sensitivity correlations between activity/expression and gene essentiality shows there are more cases where an increase in sensitivity is associated with increased expression/activity. **A, C, E,** ARACNe regulons. **B, D F,** GRNdb-like regulons.

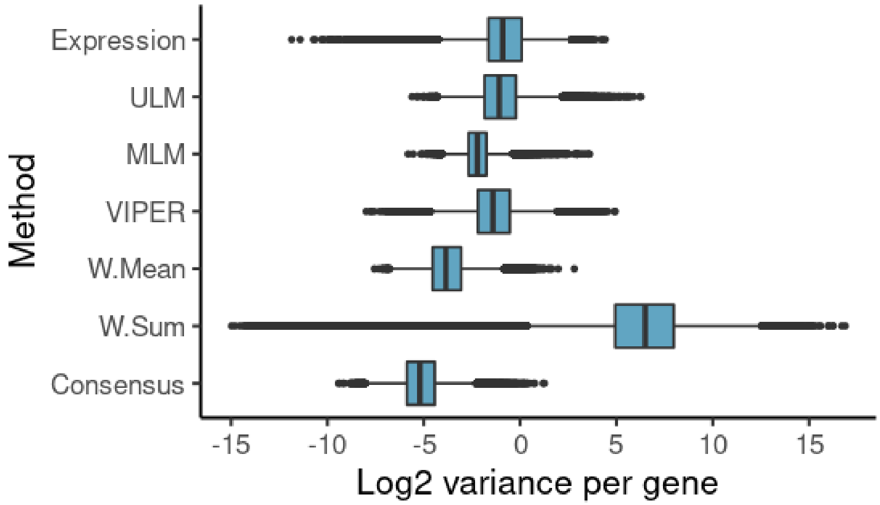

Supplementary Fig. 9 | Per gene variance for each method, across all regulon sources. Boxplots showing the log2 variance for each activity method and expression. Black line shows median; blue box represents the interquartile range; black dots show outliers.

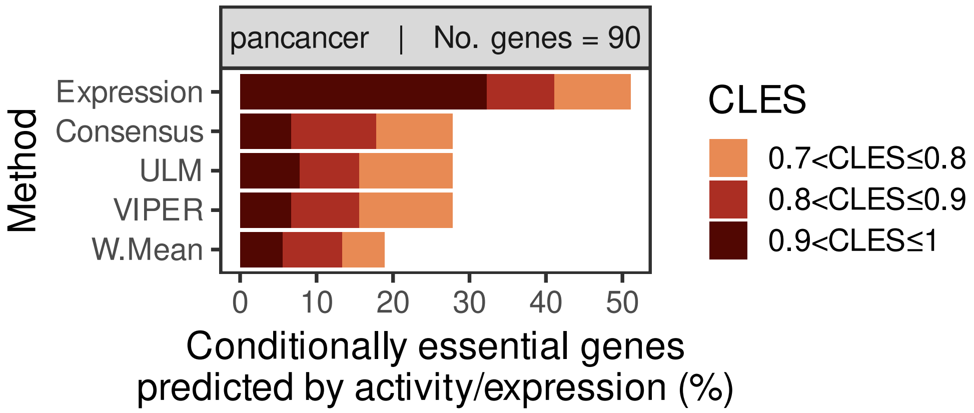

Supplementary Fig. 10 | Gene essentiality correlates better with expression than with inferred activity using DoRothEA regulons across in a pan-cancer analysis. CLES between activity/expression and binary gene essentiality stratified incrementally from CLES = 0.7 to 1 to show the percentage of significant conditionally essential genes predicted by activity/expression ($p < 0.05$) after filtering out genes that are not essential and genes that are essential in less than three cell lines in a cancer type. Methods are sorted top-to-bottom in order of performance across all cancer types for the GRN-inferred method in cause.

Unpaired Two-Samples Wilcoxon Test p-values		
Method	ARACNe	GRNdb
Consensus	0.004	<0.001
ULM	0.006	<0.001
MLM	0.206	0.002
W. Mean	0.008	<0.001
W.Sum	0.008	<0.001
VIPER	0.011	0.003

Supplementary Table 1 | P-value table of the unpaired two-samples Wilcoxon test comparing the ranks of the activity vs essentiality absolute correlation between cancer type-matched and cancer type-mismatched regulons for each activity method. (Same as in Fig 3A, B).
