# Supplementary material for: Gene essentiality in cancer is better predicted by mRNA abundance than by gene regulatory network-inferred activity
Source: NAR Cancer. 2023 Nov 28;5(4):zcad056. doi: 10.1093/narcan/zcad056 (PMC10683780; doi:10.1093/narcan/zcad056)
Supplement: zcad056_Supplemental_Files [file zcad056_supplemental_files.zip › Supplementary_tables_captions.docx]

**Supplementary tables captions**

**Supplementary Table 2 | Pearson’s correlations coefficients and p-values for correlations between activity/expression and sensitivity to inhibition for ARACNe, DoRothEA and GRNdb, ARACNe CCLE and GRNdb-like**

**Supplementary Table 3 | Enriched Gene Ontology analysis terms for genes with a correlation > 0.6 for ARACNe, GRNdb and mRNA expression**

**Supplementary Table 4 | CLES coefficients and Wilcoxon test p-values for testing conditionally essential genes for ARACNe, GRNdb and DoRothEA**
